# Interactome Analysis of Tau‐seed Isolated from AD Brains Suggests New Mechanism for Tau Aggregation and Spreading

**DOI:** 10.1002/alz.091621

**Published:** 2025-01-03

**Authors:** Pablo Martinez, Yanwen You, Henika Patel, Nur Jury, Yuhao Min, Javier Redding, Xiaoqing Huang, Sayan Dutta, Amber L. Mosley, Jean‐Christophe Rochet, Jie Zhang, Nilüfer Ertekin‐Taner, Juan C Troncoso, Cristian A Lasagna Reeves

**Affiliations:** ^1^ Department of Anatomy, Cell Biology, and Physiology, Indiana University School of Medicine, Indianapolis, IN USA; ^2^ Stark Neurosciences Research Institute, Indiana University School of Medicine, Indianapolis, IN USA; ^3^ Mayo Clinic, Jacksonville, FL USA; ^4^ Johns Hopkins University School of Medicine, Baltimore, MD USA; ^5^ 14482 Carlow Run, 14482 Carlow Run, IN USA; ^6^ Purdue Inst. for Integrative Neurosci., Purdue University, Lafayette, IN USA; ^7^ Dep. of Medicinal Chemistry and Molecular Pharmacology, Purdue University, Lafayette, IN USA; ^8^ Dep. of Biochemistry and Mol. Biology, Indiana Univ. Sch. of Med, Indianapolis, IN USA; ^9^ Center for Comp. Biology and Bioinformatics, Indiana University, School of Medicine, Indianapolis, IN USA; ^10^ Department of Medical and Molecular Genetics, Indiana University, School of Medicine, Indianapolis, IN USA; ^11^ Center for Computational Biology and Bioinformatics, Indiana University School of Medicine, Indianapolis, IN USA

## Abstract

**Background:**

Tau aggregates, a hallmark of Alzheimer’s disease (AD) and other tauopathies, spread throughout the brain, contributing to neurodegeneration. How this propagation occurs remains elusive. Previous research suggests that tau‐seed interactors play a crucial role. Based on this, the study aimed to identify novel tau‐seed interactors in AD brains and validate their impact in vivo.

**Method:**

AD and control brain extracts were separated in fractions by Size Exclusion Chromatography. Fractions with the highest tau seeding activity, measured using a tai‐biosensor cell line, were analyzed by mass spectrometry to identify interacting proteins. Bioinformatic tools dissected enriched pathways, identifying interactors that were validated in a Drosophila tauopathy model by genetically interfering with their homologs and assessing tau accumulation and eye degeneration.

**Results:**

Tau seeding activity was concentrated in high molecular weight fractions containing only a small portion of total tau in the AD brains. Compared to controls, AD brains revealed a distinct interactome for tau‐seeds, enriched in proteins associated with synaptic and mitochondrial pathways. Notably, Drosophila screening confirmed that several novel interactors significantly reduced tau accumulation and eye degeneration, suggesting their potential therapeutic relevance.

**Conclusion:**

This study sheds light on tau propagation mechanisms in AD by identifying novel tau‐seed interactors. These interactors, particularly those involved in synaptic and mitochondrial pathways, offer promising targets for therapeutic interventions aimed at decreasing tau spread and potentially preventing neurodegeneration in tauopathies. The findings add to the growing evidence that targeting tau‐seed interactors, like previously identified BSN, could represent a novel strategy for treating these debilitating conditions.

